# Resistance patterns of multidrug resistant *Acinetobacter baumannii* in an ICU of a tertiary care hospital, Malaysia

**DOI:** 10.12669/pjms.316.8445

**Published:** 2015

**Authors:** Sivakami Janahiraman, Muhammad Nazri Aziz, Fan Kee Hoo, Hon Shen P’ng, Yang Liang Boo, Vasudevan Ramachandran, Ahmad Fuad Shamsuddin

**Affiliations:** 1 Sivakami Janahiraman, Faculty of Pharmacy, Universiti Kebangsaan Malaysia, Wilayah Persekutuan Kuala Lumpur, Malaysia; 2 Muhammad Nazri Aziz, LABLINK (M) SDN. BHD. Bangunan LABLINK, 14 (129) Jalan Pahang Barat, Off Jalan Pahang, 53000 Kuala Lumpur. Malaysia; 3 Fan Kee Hoo, Department of Medicine, Faculty of Medicine and Health Sciences, Universiti Putra Malaysia, Serdang, Selangor, Malaysia; 4 Hon Shen P’ng, Department of Medicine, Hospital Sultanah Nora Ismail, Batu Pahat, Johor, Malaysia; 5 Yang Liang Boo, Department of Medicine, Hospital Enche’ Besar Hajjah Khalsom, Kluang Johor, Malaysia; 6 Vasudevan Ramachandran, Malaysian Research Institute on Ageing, Universiti Putra Malaysia, Serdang, Malaysia; 7 Ahmad Fuad Shamsuddin, Faculty of Pharmacy, Universiti Kebangsaan Malaysia, Wilayah Persekutuan Kuala Lumpur, Malaysia

**Keywords:** Ventilator-associated pneumonia, *Acinetobacter baumannii*, Drug Resistance, Intensive Care Units, Malaysia, *Acinetobacter baumannii*: ACB, intensive care units: ICU, multidrug-resistant: MDR, ventilator-associated pneumonia: VAP, Clinical and Laboratory Standards Institute: (CLSI), Culture and sensitivity: C&S

## Abstract

**Backgrounds & Objective:**

Antimicrobial resistance is a major health problem worldwide in hospitals. The main contributing factors are exposures to broad-spectrum antimicrobials and cross-infections. Understanding the extent and type of antimicrobial use in tertiary care hospitals will aid in developing national antimicrobial stewardship priorities.

**Methods:**

In this study, we have analyzed the antimicrobial agents’ usage for acquisition of multidrug resistant using retrospective, cross-sectional, single-centre study in a multidisciplinary ICU at tertiary care hospital.

**Results:**

*Acinetobacter baumannii* (ACB) was isolated in various specimens from 662 patients. From these, 136 patients who were diagnosed with Ventilator-associated pneumonia (VAP) caused by ACB were included into the study. In our study, MDR strain accounts for 51% of all VAP cases caused by ACB. The development of ACB VAP were 10.5 + 6.4 days for MDR strains compared to susceptible organism (7.8 + 4.5 days) and had significantly longer ICU stay.

**Conclusion:**

The study concludes that prudent use of antimicrobial agents is important to reduce acquisition of MDR ACB.

## INTRODUCTION

Antimicrobial resistance is a serious global threat in combating infectious diseases. Infections caused by resistant pathogens result in significant morbidity and mortality, as well as incur significant healthcare costs worldwide.^1^ Among these pathogens, *Acinetobacter baumannii* (ACB) has the propensity to cause a wide spectrum of opportunistic infections.^2^ With its ability to develop novel mechanism of resistance during treatment coupled with intrinsic resistance to numerous antimicrobial agents, ACB poses major challenges for healthcare management worldwide.^3^

Patients in intensive care units (ICU) are at the greatest risk of acquiring these multidrug resistant (MDR) organisms owing to multiple factors such as importation of resistant microorganism at admission, extensive use of broad-spectrum antimicrobial agents, and cross transmission from one patient to another.^4^ The rising incidence of MDR ACB may be attributed to lack of infection-control measures and high selective pressure of commonly used antimicrobial agents.^5^

In Malaysia, the prevalence of *Acinetobacter spp* isolates increased by two-fold from year 2006 to year 2007.^6^ However, local data on factors affecting development of MDR ACB is lacking. Thus, this study was designed to evaluate the risk factors, especially antimicrobial agents’ usage, for acquisition of MDR ACB as a causative agent for ventilator-associated pneumonia (VAP).

## METHODS

We conducted a retrospective, cross-sectional,single-centre study in a multidisciplinary ICU of a tertiary care hospital. In this hospital, antibiotic susceptibility testing by disc diffusion was in accordance with the Clinical and Laboratory Standards Institute (CLSI).

A convenient sampling method was employed to recruit subjects, as the precise total numbers of patients with ACB isolates were unknown to calculate sample size. Culture and sensitivity (C&S) data for selected culture-positive ACB samples were collected from medical record of adult patients (18 years old or more) admitted to ICU in 2009. All patients with ACB isolates from various specimens were initially screened. Only those with both culture-positive tracheal aspirates for ACB and clinical diagnosis of VAP were included. VAP refers to pneumonia that arises more than 48 hours after endotracheal intubation. VAP was clinically diagnosed based on a new or worsening pulmonary infiltrate associated with at least two out of four signs of infection (pyrexia above 38.5ºC, leukocytosis, purulent sputum, crepitations in lungs or gas exchange imbalance) developed in patients on mechanical ventilation.^7^

Demographic data, admission history, and prior antimicrobial exposure (including all antimicrobial agents preceding ACB VAP infection from the day of hospital admission) were extracted from patient’s medical record. Antimicrobial sensitivity of ACB isolates was reviewed. Antimicrobial resistance was reported as the percentage of isolates per month. MDR ACB is defined as ACB that is resistant to more than two of the following five drug classes of antipseudomonal cephalosporins, carbapenems, β-lactam/β-lactamase inhibitors combination, fluoroquinolone or aminoglycosides.

Statistical analysis was performed using IBM SPSS version 16.0. Categorical variables were expressed as numbers and percentages while continuous variables were expressed as mean ± standard deviation. Independent t-test was used for comparison of continuous variables and chi-square test was used to compare categorical variables. Binary logistic regression analysis was employed to determine independent risk factors. Odds ratio (OR) and 95% confidence interval were reported where appropriate. Differences between groups were considered significant if the p value was < 0.05.

## RESULTS

**Fig.1:**
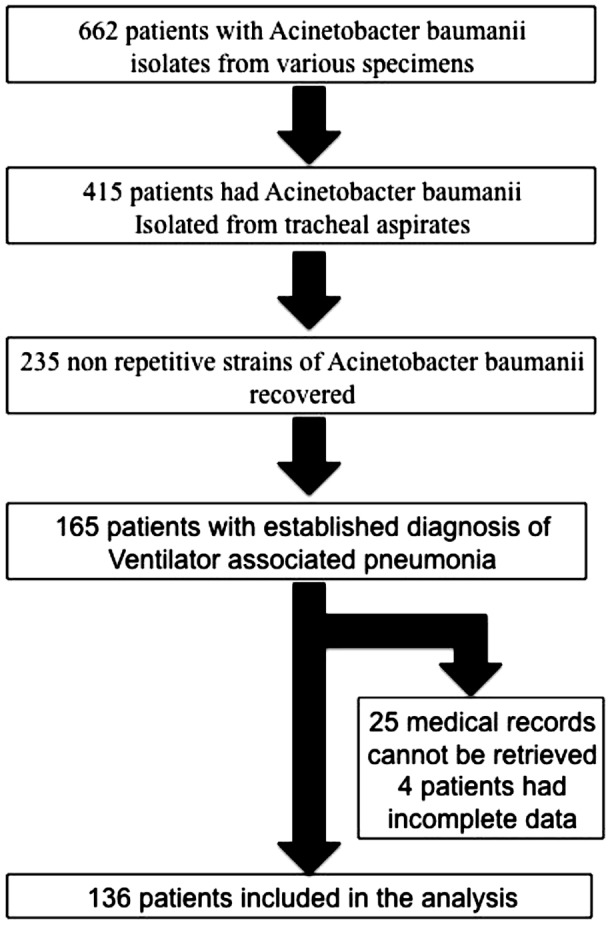
Flow chart showing number of patients who met the inclusion criteria and recruited into the final analysis.

ACB was isolated in various specimens from 662 patients. From these, 136 patients who were diagnosed with VAP caused by ACB were included into the study (Fig.1), of which 69 patients had MDR isolates, and 67 patients had susceptible isolates.

Characteristics of patients who developed ACB VAP are shown in Table-I. Majority of patients were men (67%) with mean age of 47 ± 18 years. The later the onset of VAP in ICU, the more likely the causative agent is of MDR strain. Logistics regression analysis showed odds of 1.014 for acquiring MDR strain, for every one-day of ICU stay prior to infection. Total duration of ICU stay is also a risk factor for MDR strains, with 3.7% increase in odds for every extra day of total ICU stay. Patients who underwent surgery were 2.19 times more likely to acquire MDR strains as causative agents for VAP. Gender, age, ethnicity and total hospital stay do not affect the odds of acquiring MDR ACB.

**Table-I:** Demographic data of patients with MDR ACB VAP infection compared to susceptible ACB VAP infection. Bold signifies p<0.05.

Variables	MDR Acinetobacter baumannii n= 69	Susceptible Acinetobacter baumannii n= 67	p value	OR (95% CI)
Age (years) (mean(sd))	47.4 (19.1)	47.2 (16.9)	0.930^a^	1.001 (0.982-1.020)
*Gender (freq (%))*					
Male	42.0 (60.9)	49.0 (73.1)	0.129^b^	1.0
Female	27.0 (39.1)	18.0 (26.9)		1.750 (0.848-3.613)
*Ethnic (freq(%))*					
Malay	42.0 (60.9)	36.0 (53.7)	0.177^b^	1.0
Chinese	10.0 (14.5)	19.0 (28.4)		1.750 (0.277-11.060)
Indian	15.0 (21.7)	9.0 (13.4)		0.789 (0.113-5.528)
Others	2.0 (2.9)	3.0 (4.5)		2.500 (0.348-17.941)
*Length of hospital stay*					
prior to infection (days; mean (sd))	25.7 (19.1)	15.1 (9.8)	0.061^a^	1.023 (0.967-1.002)
*Length of ICU stay*					
prior to infection (days) (mean(sd))	10.5 (6.4)	7.8 (4.5)	0.030^a^	1.014 (0.934-1.011)
Total hospital stay (days) (mean(sd))	38.5 (21.2)	32.2 (33.4)	0.210^a^	1.009 (0.995-1.024)
Total ICU stay (days) (mean(sd))	33.2 (12.5)	17.9 (12.1)	0.015^a^	1.037 (1.007-1.067)
Prior surgery (freq(%))	34 (49.3)	21 (31.3)	0.029^b^	2.190 (1.086-4.419)

a Binary logistic regression

b Crosstabs Chi-square.

**Fig.2:**
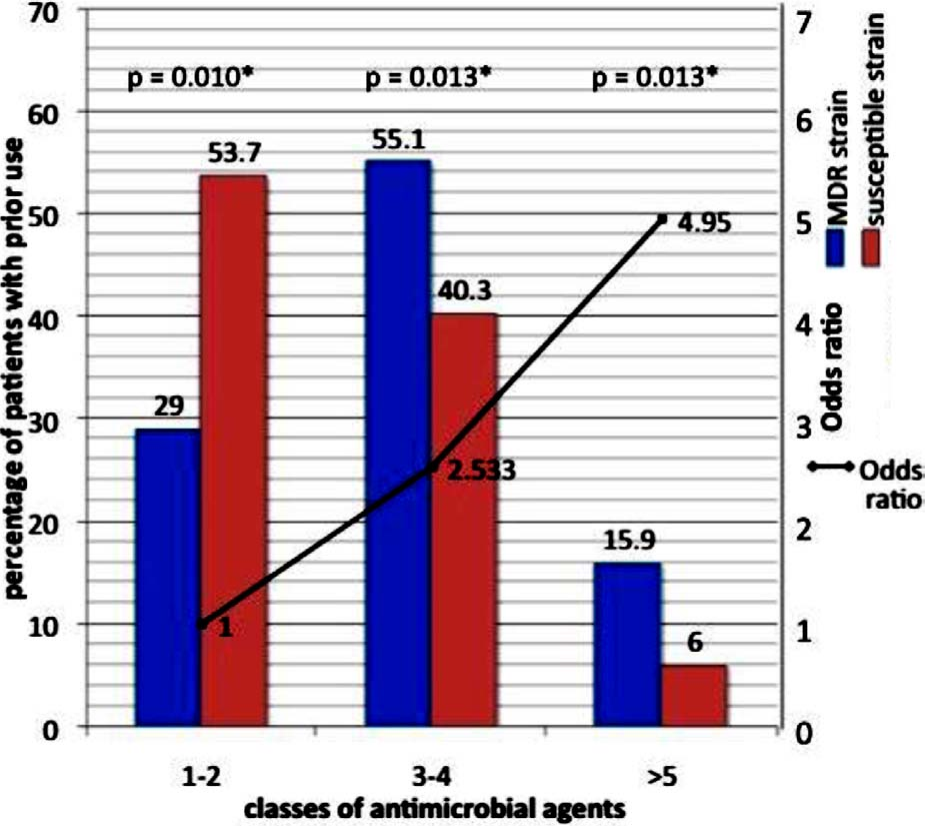
Percentage of patients with prior antimicrobial agents used in patients who developed MDR strain VAP vs susceptible strain VAP, binary logistic regression analysis for odds ratio of acquiring MDR strain infection with prior use of antibiotics (group with prior use of 1-2 antibiotics taken as standards for comparison).

Antimicrobial agents used prior to development of VAP caused by ACB are depicted in Table-II. Pearson chi-square analysis revealed that prior exposure to carbapenems and cephalosporins(both second and third generation) were associated with the acquisition of MDR strain. The odds of acquiring MDR ACB were increased by factors of 4.491 and 17.143 with the prior use of cephalosporin and carbapenem, respectively. The duration of prior antimicrobial agents used was also higher in patients with MDR strain infection (19.3 (sd. 10.5) vs 11.5 (sd. 7.5) days, binary logistic regression p value <0.001). Every one-day increase in the usage of prior antimicrobial agents heightened the risk of acquiring MDR organism by 14.1% (95% confidence interval 7.6% - 21%). The odds of acquiring MDR ACB VAP are incremental as the number of antimicrobial class increases. When exposure to one to two classes of antimicrobials is used as standards for comparison, the odds of acquiring MDR strain VAP were 2.5 times and 5 times for those with prior usage of three to four classes of antimicrobials and five or more classes of antimicrobials respectively (Fig.2).

**Table-II:** Frequency and percentage of prior exposure of antimicrobial agents in patients with MDR ACB VAP compared to susceptible ACB VAP; crosstabs Chi square analysis for odds ratio of developing MDR strain infection. Bold signifies p<0.05.

Variables	MDR Acinetobacter baumannii n = 69	Susceptible Acinetobacter baumannii n = 67	p-value	OR (95% CI)
*Classes of antimicrobials (freq(%))*				
Penicillins	23 (33.3)	15 (22.4)	0.155	1.733 (0.269-1.236)
Cephalosporins	57 (80.6)	46 (68.3)	0.016	4.491 (1.206-16.721)
Macrolides	16 (23.2)	21 (31.3)	0.285	0.661 (0.309-1.415)
Fluoroquinolones	4 (9.0)	6 (5.8)	0.481	0.626 (0.168-2.325)
Aminoglycosides	5 (7.2)	10 (14.9)	0.153	0.445 (0.144-1.380)
Glycopeptides	10 (14.5)	5 (7.5)	0.191	2.102 (0.678-6.514)
Carbapenems	46 (66.7)	7 (10.4)	<0.001	17.143 (8.769-43.412)
β-lactam/β-lactamase inhibitors, penicillins	37 (53.6)	32 (47.8)	0.494	1.265 (0.645-2.480)
Other β-lactam/β-lactamase inhibitors	12 (17.4)	9 (14.5)	0.736	0.867 (0.377-1.993)
Imidazoles	16 (23.2)	11 (16.4)	0.322	1.537 (0.654-3.612)

## DISCUSSION

At present, major concern is the increase in resistance of non-lactose fermenting Gram-negative organism not to one but multiple antimicrobial agents.*Acinetobacter spp* has gained recognition as a nosocomial pathogen with higher propensity to affect severely ill patients. Among *Acinetobacter* spp, ACB is the most represented species in VAP and known to be more resistant to antimicrobials.^8^ Whilst many patients in ICU may only be colonized, the debilitated nature of the majority of these patients renders them susceptible to the development of life-threatening infection.

This study showed that the mean duration of ICU admission to development of ACB VAP infection were 7.8 ± 4.5 days for susceptible organism, and 10.5 ± 6.4 days for MDR strains, suggesting that most patients had late-onset VAP. This is in parallel with previous studies, which reported ACB as a late-onset pathogen.^9^,^10^ Deris et al. found that patients with *Acinetobacter* bacteremia had longer ICU stay of 12.7 ± 17.3 days in comparison to patients with other gram-negative blood stream infection.^9^

MDR strain accounts for 51% of all VAP cases caused by ACB in our study. The patients who acquired MDR ACB VAP had significantly longer total ICU stay compared to those with susceptible strain in our study. Notably, other studies acknowledged prolonged stay in high-risk unit as one of the risk factors to developing MDR strains.^10^ Hence, a high frequency of MDR ACB detected in ICU indicates that longer ICU stay may promote selection of bacterial resistance to antimicrobials, perhaps due to extensive use of broad-spectrum antimicrobials together with inconsistent infection-control measures.

Based on this study, patients who underwent operation are 2.2 times more likely to acquire MDR organism compared to patients without any surgical intervention. This suggests that invasive procedure might be one of the main predisposing factors for MDR ACB infection. Surgery procedures could play a significant role in the emergence of multiresistant isolates owing to early exposure to microbials during procedure, extended use of antimicrobial prophylaxis and associated infectious complications in the postoperative period.

Prior treatment with antimicrobials contributes significantly to development of MDR strains. The prescription of penicillins, β-lactam/β-lactamase inhibitor combinations, glycopeptides and imidazoles preceding infection amidst MDR ACB VAP patients were noted to be higher than susceptible ACB VAP subjects but the difference was not statistically significant. However, prior exposures to carbapenems and cephalosporins were the risk factors significantly associated with MDR strain infection. Increased use of extended spectrum antimicrobials leads to the emergence of resistant strains by possible destruction of the normal microflora, mutation or selection of resistant strains. This finding is in agreement with other studies, which documented that prior usage of broad-spectrum antimicrobial agents increased the risk for isolation of MDR ACB.^10^,^11^ In particular, cephalosporin and carbapenem use correlates highly to subsequent infection with β-lactam and/or carbapenem resistant *Acinetobacter spp* as a consequence of collateral damage in various studies.^12^-^15^ Hence, the use of one antimicrobial agent can enhance resistance mechanisms of the organism to other antimicrobial agents.

Although the antimicrobial use has been identified as a risk factor for promoting antimicrobial resistance but only few studies had proven the association between duration of antimicrobial agent usage and development of resistant strains.^16^ There were reports that a shorter duration of antibiotic course was related with less antimicrobial resistance in patients with suspected VAP.^17^,^18^ Nevertheless, these results were in contrast with that of Donaldson et al. findings wherein they were unable to prove that duration of antimicrobial therapy was associated to the emergence of resistance organisms in a large prospective observational study of 415 critically ill patients.^16^ Our study agrees on a significant association between duration of antimicrobial exposure and nosocomial MDR ACB infection development. Not surprisingly, our study also illustrated that intense treatment with a higher number of antimicrobial agents in severely ill patients predisposes to MDR strain infection, more likely suggestive of selection pressure, consistent with the results in the study by Jung et al.^19^

Generalized application of the study result is limited owing to the study design with all inherent problems. This study did not attempt to assess the colonization pressure by conducting molecular typing. Other factors contributing to antimicrobial resistance namely, inappropriate dosing, delay in treatment, methods of administration and distribution of drug to the site of infection, other invasive procedures and patients’ co-morbid conditions were not investigated in this study. Determination of minimum inhibitory concentration values for each antibiotic was unavailable, as it was not done during the study period.

## CONCLUSION

Risk factors for acquiring MDR ACB VAP are longer stay in ICU, duration of ICU stay prior to the onset of VAP, having surgical intervention, prior receipt of carbapenem and cephalosporins, longer duration of prior antimicrobial therapy and higher number of antimicrobial use. This study serves as a reminder that prudent use of antimicrobial agents is important to reduce acquisition of MDR ACB.
